# Acknowledgment of Reviewers

**DOI:** 10.1002/aps3.11408

**Published:** 2021-01-21

**Authors:** Beth Parada

The editors gratefully acknowledge our reviewers, who have generously given their time and expertise to review manuscripts submitted to *Applications in Plant Sciences*. The list includes those who reviewed manuscripts from December 30, 2019, to December 30, 2020. Thank you for helping *APPS* maintain a prompt and fair peer‐review process.

Amarasinghe, Prabha

Anvarkhah, Sepideh

Arrington, Matthew

Baldrich, Patricia

Barroso de Morais, Érica

Bianconi, Andre

Blackstock, Joshua

Bonnet, Pierre

Braukmann, Thomas

Brown, Herrick

Cassab, Gladys

Castro, Pedro Humberto

Chambers, Alan

Chapman, Mark

Chau, John

Clarke, Jennifer

Dai, Qi

Dikow, Rebecca

Doyle, Jeffrey

Ellwood, Elizabeth

Farrar, Kerrie

Fay, Michael

Fazio, Gennaro

Flachowsky, Henryk

Fonseca, Jose

Gage, Joseph

Gehan, Malia

Giri, Jitender

Graves, Sarah

Halling, Roy

Harbert, Robert

Harris, Jesse

Hearn, David

Hepler, Nathan

Herrando‐Moraira, Sonia

Hileman, Lena

Hodel, Richard

Hosseinalizadeh Nobarinezhad, Mahboubeh

Huang, Daisie

Huang, Hui

Inglada, Jordi

Ison, Jennifer

Jantzen, Johanna

Jellen, Eric

Johnson, Leigh

Jordon‐Thaden, Ingrid

Joshi, Dinesh

Kates, Heather Rose

Kenchanmane Raju, Sunil Kumar

Khaki, Saeed

Kim, Sangtae

Koptur, Suzanne

Kraichak, Ekaphan

Kuzmina, Maria

Lahiri, Sriyanka

Leebens‐Mack, James

Liang, Zhikai

Liu, Yang

Livingston, Sam

Lobet, Guillaume

Losada, Juan

Lyu, Shan‐hua

Majure, Lucas

Mandel, Jennifer

Marconi, Sergio

Marsico, Travis

Marx, Hannah

Mason, Chase

Mast, Austin

Mavrodiev, Evgeny

Maya‐Lastra, Carlos

McKain, Michael

Meerdink, Susan

Miller, Nathan

Morales‐Briones, Diego

Morris, Daniel

Motivans, Elena

Mwebaze, Ernest

Neubig, Kurt

Nguyen, Van Sinh

Nikkeshi, Aoi

Ogutcen, Ezgi

Olson, Eric

Orli, Sylvia

Pandey, Bipin Kumar

Park, Daniel

Pasiche‐Lisboa, Carlos

Pearson, Katelin

Pec, Gregory

Pinson, Jerald

Pokorny, Lisa

Priya, Piyush

Pryer, Kathleen

Rahmatpour, Nasim

Ridger, Perry

Robbins, Matthew

Roberts, Joe

Ruiz‐Muñoz, Jose

Santos, Thiago

Shin, Junha

Singh, Arti

Sinharoy, Senjuti

Smith, Matthew

Spalding, Edgar

Stavness, Ian

Stucky, Brian

Sutherland, Brittany

Sweeney, Patrick

Tomasi, Carlo

Topp, Christopher

Tunison, Robert

Tzanetakis, Ioannis

Unruh, Sarah

Valderrama Escallón, Eugenio

Wang, Jinpeng

Weitemier, Kevin

Weng, Xiaoyu

Yang, Ya

York, Larry

Yost, Jenn

Zhang, Mingchu

